# Long-Term Efficacy of Mitral Valve Transcatheter Edge-to-Edge Repair (M-TEER) for Exercise-Induced Mitral Regurgitation in a Cardiac Resynchronization Therapy (CRT) Non-responder: A Three-Year Follow-Up

**DOI:** 10.7759/cureus.71793

**Published:** 2024-10-18

**Authors:** Hiroto Yagasaki, Takeki Suzuki, Keitaro Watanabe, Yoshitake Oshima, Toshiyuki Noda

**Affiliations:** 1 Cardiology, Gifu Prefectural General Medical Center, Gifu, JPN; 2 Medicine, Indiana University School of Medicine, Indianapolis, USA

**Keywords:** cardiac resynchronization therapy (crt), exercise stress echocardiography, heart failure, mitral valve regurgitation, mitral valve transcatheter edge-to-edge repair

## Abstract

Mitral valve transcatheter edge-to-edge repair (M-TEER), a minimally invasive procedure that uses a clip to join the mitral valve leaflets, has emerged as an established treatment for severe mitral regurgitation (MR) in drug-refractory heart failure (HF). This case report presents an 80-year-old cardiac resynchronization therapy (CRT) non-responder with a complex cardiac history who underwent successful M-TEER. Despite optimal therapy, including CRT, she experienced recurrent HF symptoms. While resting echocardiography showed mild MR, exercise stress echocardiography (ESE) revealed severe MR. The M-TEER procedure resulted in trivial residual MR and significant symptom improvement. The patient's New York Heart Association (NYHA) functional class improved from III to I, with sustained benefits for three years post procedure. This case highlights the importance of comprehensive MR assessment, including ESE, in complex scenarios. It also underscores the potential long-term benefits of M-TEER in carefully selected CRT non-responders, even with borderline right ventricular function, when supported by thorough multidisciplinary evaluation.

## Introduction

Mitral valve transcatheter edge-to-edge repair (M-TEER) with the MitraClip® (Abbott Vascular, Santa Clara, CA, USA) has emerged as an established treatment for patients with severe mitral regurgitation (MR) and drug-refractory heart failure with reduced ejection fraction (HFrEF) [1]. Cardiac resynchronization therapy (CRT) effectively manages patients with HFrEF with left bundle branch block in many patients, but 30-40% do not respond adequately [2]. Recent studies have shown potential benefits of M-TEER in CRT non-responders, but long-term outcomes in this complex patient population remain unclear [3, 4]. The dynamic nature of MR can lead to underestimation of its severity at rest. Exercise stress echocardiography (ESE) has proven valuable for comprehensively evaluating MR, potentially revealing exercise-induced regurgitation not apparent at rest [5]. However, the efficacy of M-TEER for CRT non-responders with exercise-induced severe MR, especially when guided by ESE findings, remains to be fully elucidated. This case report describes a CRT non-responder with a complex cardiac history who underwent successful M-TEER for severe MR detected only during ESE. 

## Case presentation

An 80-year-old woman presented to the hospital with acute decompensated heart failure (HF). Her medical history included myocardial infarction resulting in ventricular septal rupture (VSR), complete atrioventricular block treated with a pacemaker, mild to moderate aortic regurgitation, chronic kidney disease, type 2 diabetes, and hypertension. Her medications included pitavastatin, telmisartan/amlodipine, bisoprolol, azosemide, tolvaptan, enalapril, pimobendan, and empagliflozin. Four years prior, the patient experienced a myocardial infarction in the distal right coronary artery (segment 3), leading to VSR. She received a drug-eluting stent and surgical VSR repair. Complete atrioventricular block developed post-surgery, requiring dual chamber pacemaker implantation. Left ventricular ejection fraction (LVEF) after pacemaker implantation was 42%.

**Figure 1:**
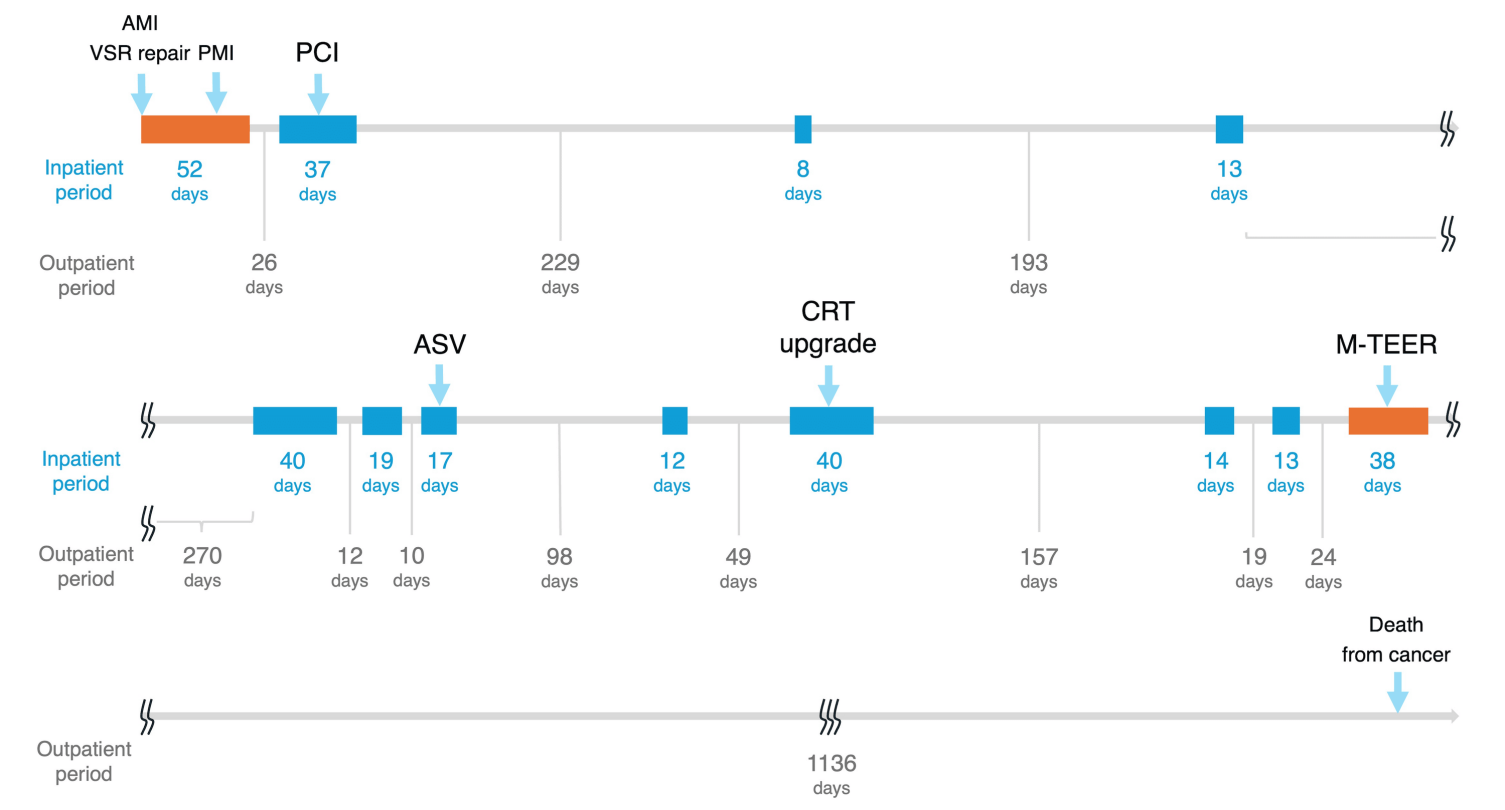
A timeline of the patient's treatment events and heart failure hospitalizations Blue boxes: heart failure hospitalizations; orange boxes: initial and current admissions AMI: acute myocardial infarction; VSR: ventricular septal rupture; PMI: pacemaker implantation; PCI: percutaneous coronary intervention; ASV: adaptive service ventilation; CRT: cardiac resynchronization therapy; M-TEER: mitral valve transcatheter edge-to-edge repair

One month post discharge, the patient was readmitted for HF exacerbation, suspected to be ischemia-related. Two drug-eluting stents were placed in the proximal and mid-left anterior descending artery (segments 6-7). Subsequently, due to poor adherence to medication and dietary restrictions, she experienced multiple hospitalizations for HF, with increasingly shorter intervals between admissions (Figure 1). During her sixth admission, she started adaptive servo-ventilation (ASV) for central sleep apnea, resulting in a three-month HF-free period. On her eighth admission, her LVEF decreased to 25% with a QRS duration of 180-200 ms, even with optimized medical therapy. The device achieved over 99% biventricular pacing, even during episodes of atrial fibrillation. However, despite this high percentage of biventricular pacing, this intervention did not improve the QRS duration, left ventricular volumes, LVEF, and New York Heart Association (NYHA) functional class III symptoms, which all remained largely unchanged. Despite improved medication adherence, ASV compliance, and sodium restriction, NYHA class II-III symptoms persisted with frequent HF exacerbations. The current admission marked her eleventh HF hospitalization. 

On presentation, the patient had hypertension (170/60 mmHg), tachycardia (110 beats per minute), tachypnea (24/min), and hypoxia (oxygen saturation (SpO_2_) 72% on room air). Examination revealed jugular venous distension and lung crackles. Heart sounds were obscured by respiratory noise. Lower extremities were cool without edema. Chest radiography showed a cardiothoracic ratio of 58%, bilateral pleural effusions, and pulmonary vascular congestion. An electrocardiogram demonstrated atrial fibrillation with ventricular pacemaker rhythm at 88 beats per minute. Pacemaker interrogation revealed paroxysmal atrial fibrillation episodes every three to six months. Laboratory tests indicated elevated B-type natriuretic peptide (BNP) (1441 pg/mL), creatinine (1.25 mg/dL), and an estimated glomerular filtration rate of 31.9 mL/min/1.73 m². Echocardiography revealed asymmetric contraction, diffuse hypokinesis (particularly severe to akinesis in the septal and inferior walls), mild to moderate aortic regurgitation, and MR. Initial treatment included non-invasive positive pressure ventilation, carperitide, intravenous loop diuretics, and oral spironolactone. The patient's condition stabilized, allowing her to begin ambulatory rehabilitation on day five. She tolerated short corridor walks but remained in NYHA functional class II.

**Figure 2:**
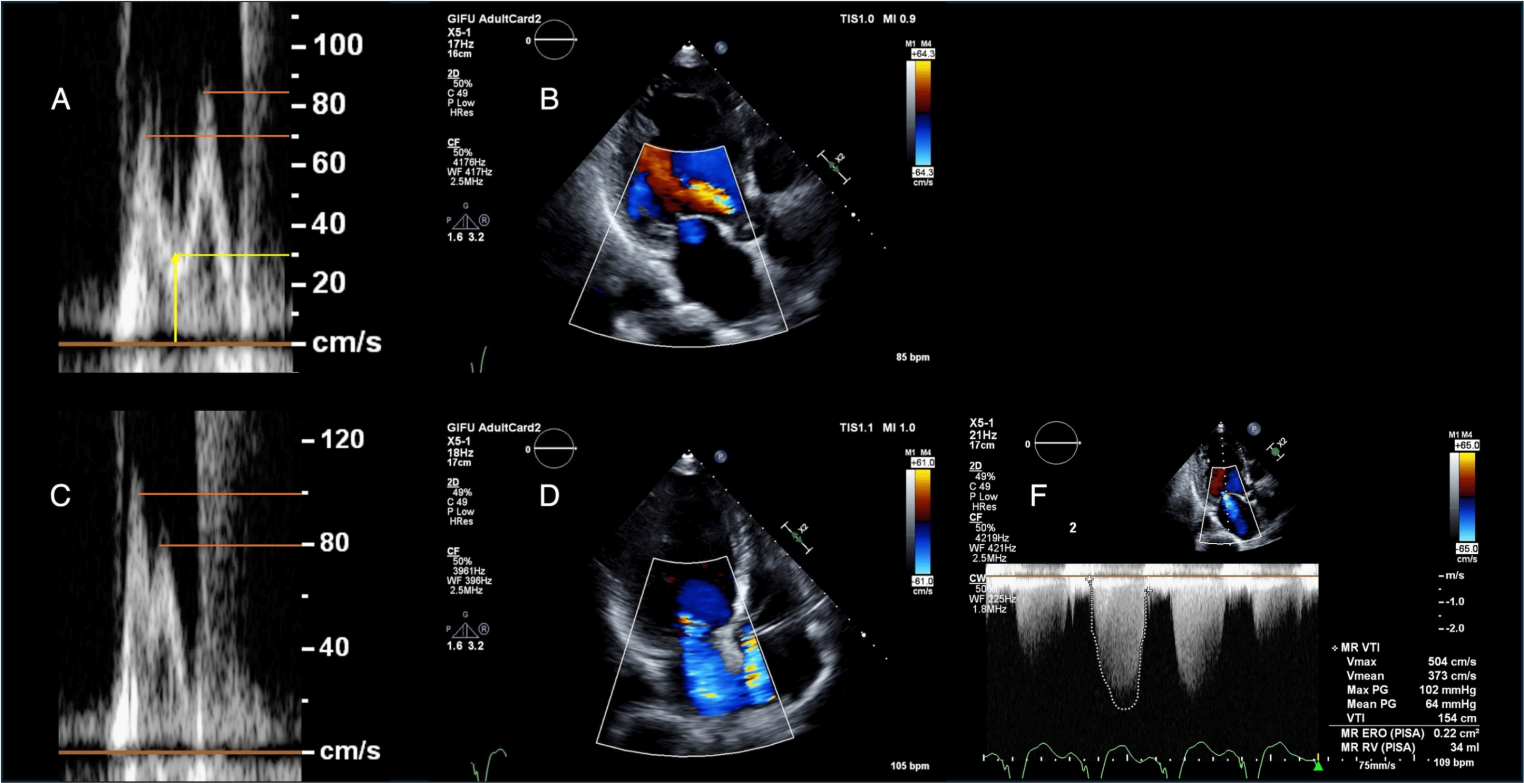
Preoperative exercise echocardiography A: E and A wave velocities at rest; E wave: 70 cm/s; A wave: 85 cm/s, initial E/A ratio: 0.82, adjusted E/A ratio: 1.27, accounting for A wave onset at 30 cm/s (yellow line); B: trivial mitral regurgitation at rest; C: E and A wave velocities at 12.5 watts; D: severe mitral regurgitation at 12.5 watts; E: quantification of mitral regurgitation at 12.5 watts.

On hospital day 12, ESE using a bicycle ergometer was performed to assess the dynamic nature of MR. At rest, mild MR was noted with unmeasurable effective regurgitant orifice area, blood pressure of 126/72 mmHg, heart rate of 87 beats per minute, E/A ratio of 0.82 (Figures 2A, 2B, Video 1), tricuspid regurgitation pressure gradient (TRPG) of 44 mmHg, and tricuspid annular plane systolic excursion (TAPSE) of 16.7 mm. At 12.5 watts, MR worsened significantly between A2 and P2 scallops, with blood pressure of 135/79 mmHg, heart rate of 112 beats per minute, effective regurgitant orifice area 0.22 cm², regurgitant volume 34 mL, E/A 1.27 (Figures 2C-2E, Video 1), and TRPG 67 mmHg (pulmonary artery systolic pressure 77 mmHg), accompanied by marked dyspnea and wheezing. Myocardial perfusion imaging using technetium-99m tetrofosmin with adenosine stress revealed no clinically significant reversible ischemia warranting revascularization. Right heart catheterization revealed a pulmonary capillary wedge pressure of 24 mmHg with prominent v waves (34 mmHg), mean pulmonary artery pressure of 35 mmHg, and cardiac index of 2.4 L/min/m², despite substantial fluid removal. 

**Video 1:** Preoperative exercise echocardiography

**Figure 3:**
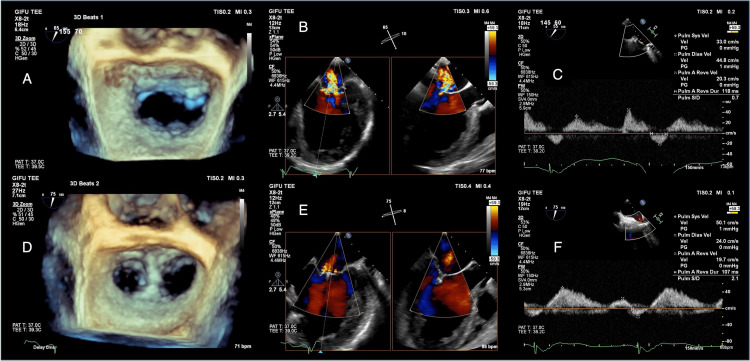
Perioperative transesophageal echocardiography A: pre-procedural three-dimensional mitral valve; B: pre-procedural two-dimensional mitral regurgitation; C: pre-procedural pulmonary vein flow; D: post-procedural three-dimensional mitral valve; E: post-procedural two-dimensional mitral regurgitation; F: post-procedural pulmonary vein flow

**Figure 4:**
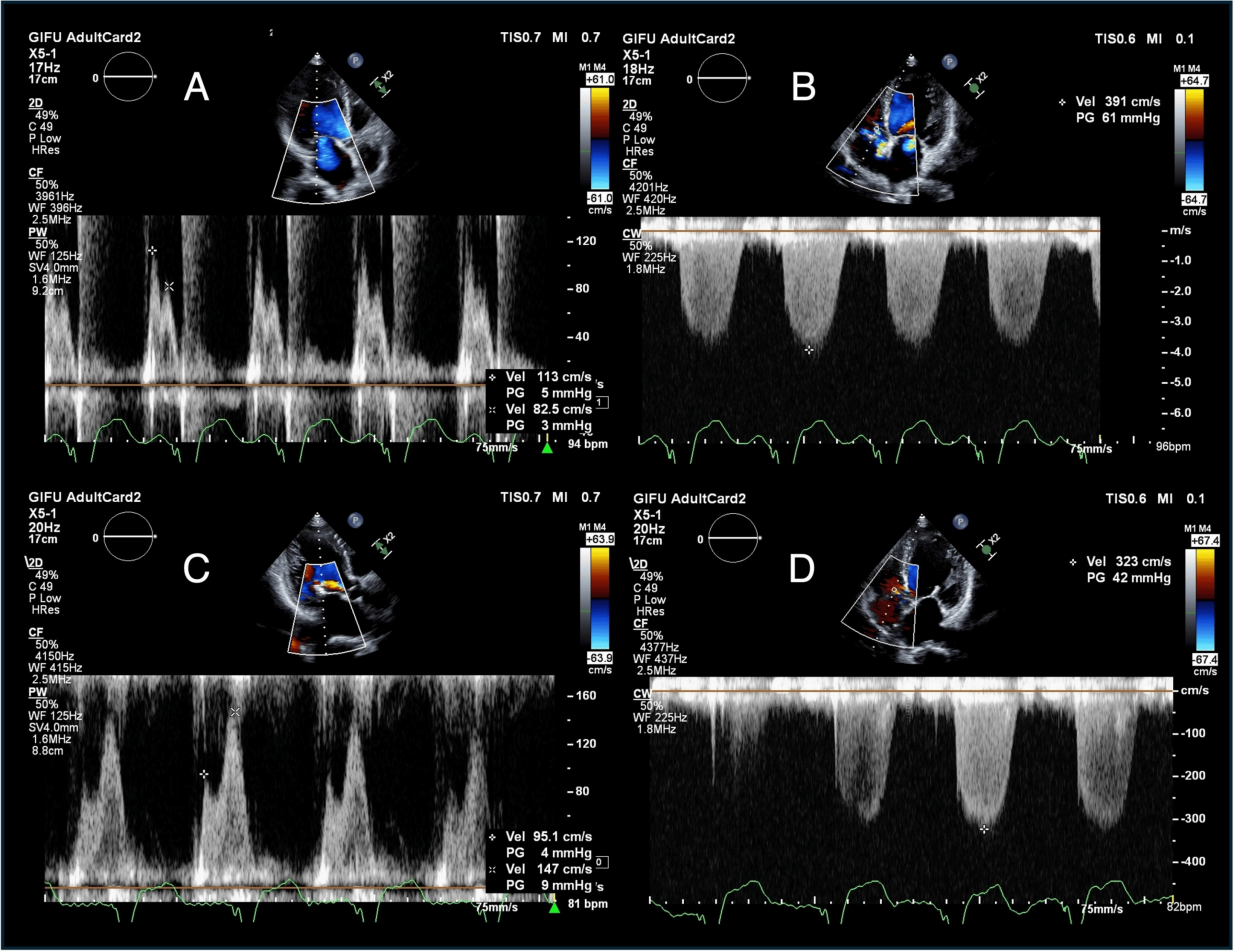
Exercise stress echocardiography at three minutes post-exercise, pre- and post-treatment Pre-treatment: A: transmitral flow (E/A 1.4); B: TRPG 61 mmHg, vitals: BP 126/70 mmHg, HR 96 bpm; Post-treatment: C: transmitral flow (E/A 0.65); D: TRPG 42 mmHg, vitals: BP 112/59 mmHg, HR 80 bpm At peak exercise post treatment, the patient exhibited E/A 1.0, TRPG 57 mmHg (PASP 67 mmHg), and moderate MR, indicating exercise-induced pulmonary hypertension. However, as shown, hemodynamic recovery was faster post-treatment. TRPG: tricuspid regurgitation pressure gradient; PASP: pulmonary artery systolic pressure; MR: mitral regurgitation; BP: blood pressure; HR: heart rate; bpm: beats per minute

Given the patient's severe dynamic MR with persistent symptoms despite optimal medical therapy, advanced age, and previous thoracotomy, our multidisciplinary heart team recommended M-TEER as the most suitable treatment approach. The M-TEER procedure was performed under general anesthesia. Although only mild MR could be reproduced under anesthesia even with vasopressors (Figures 3A, 3B), based on the ESE findings, a single MitraClip NT clip device was placed at the center between A2 and P2 scallops (Figure 3D, Video 2), where MR was most severe during ESE. This resulted in trivial residual MR (Figure 3E, Video 2). Despite the apparently minor change in MR, pulmonary vein flow increased markedly from 0.7 to 2.1 m/s (Figures 3C, 3F). The patient recovered without complications and was discharged 11 days post-procedure with a TRPG of 34 mmHg. At the one-month follow-up, the patient reported significant improvement in dyspnea, with NYHA functional class improving to I. Follow-up ESE demonstrated improved immediate post-exercise hemodynamic recovery compared to pre-treatment, potentially explaining the symptom improvement (Figures 4A-4D). The patient maintained NYHA class I, experienced no further HF hospitalizations, and had stable BNP levels for the following three years, until her death from small intestine cancer (Figure 1). 

**Video 2:** Perioperative transesophageal echocardiography

## Discussion

This case highlights the potential long-term benefits of M-TEER in carefully selected CRT non-responders with exercise-induced severe MR, even when resting evaluations may underestimate MR severity. The patient's complex cardiac history, including myocardial infarction with VSR and complete atrioventricular block, further underscores the significance of this successful intervention.

The severity assessment of MR is complex due to its dynamic nature, with left ventricular and left atrial pressure gradients significantly influencing MR behavior [5]. Transmitral flow patterns, particularly the E/A ratio, are commonly used to estimate left ventricular diastolic function and left atrial pressure [6]. With aging, diastolic relaxation typically declines, resulting in an E/A ratio <1 around age 60, even without elevated left atrial pressure [6]. As both relaxation and compliance worsen, left ventricular end-diastolic pressure rises, increasing left atrial afterload during atrial contraction. This can lead to a pseudonormal pattern (E/A ≥1) due to elevated E wave velocities [6]. In significant left ventricular relaxation abnormalities or bundle branch blocks, E and A waves may merge, shortening diastolic filling time and elevating left atrial pressure. Importantly, when the E wave velocity at the onset of the A wave exceeds 20 cm/s, subtracting this residual E wave velocity from the A wave when interpreting the E/A ratio has been proposed [6].

In our case, the resting transmitral flow showed an E wave of 70 cm/s and an A wave of 85 cm/s (E/A 0.82) (Figure 2A). However, considering the flow velocity at the A wave onset (approximately 30 cm/s), the adjusted E/A ratio becomes 1.27 [70 / (85 - 30)], suggesting a pseudonormal pattern and potential left atrial pressure elevation (Figure 2C). This elevated pressure likely contributed to the underestimation of MR severity at rest in our patient, with only trivial MR observed initially.

The dynamic nature of MR during physical exertion underscores the importance of comprehensive assessment beyond resting conditions [7, 8]. Exercise stress echocardiography (ESE) has proven valuable for evaluating MR under physiological stress, with its role in guiding treatment decisions, particularly for M-TEER in complex cases like CRT non-responders, increasingly recognized.

In our case, despite the patient's frailty, we successfully performed a low-volume ergometer-based ESE. This approach revealed severe MR at low workload, not apparent at rest, which proved crucial in guiding our decision to proceed with M-TEER. The exacerbation of MR during exercise is likely attributed to the combined effects of increased preload, afterload, and heart rate, leading to impaired left ventricular diastolic function [7,8].

Although ergometer-based testing may be challenging for frail or elderly patients often considered for M-TEER, alternative methods such as handgrip exercises have been reported [9]. However, handgrip exercises, being static upper-body exertions, primarily increase afterload with minimal effect on preload. In contrast, ergometer-based exercises engage the whole body, altering both afterload and preload significantly. Studies have shown that ergometer-based ESE is superior to handgrip exercises in predicting outcomes for patients with MR [8].

This observation highlights the complex interplay between exercise-induced cardiovascular changes and MR severity, emphasizing the value of appropriate stress testing in accurately assessing MR in certain patients.

Recent studies have shown that M-TEER can be effective for exercise-induced severe MR, even when MR is only moderate at rest [10]. Patients treated with M-TEER have demonstrated better outcomes compared to those managed with medical therapy alone [11]. Our case aligns with and extends these findings, demonstrating sustained benefits over three years in a CRT non-responder with exercise-induced severe MR. This long-term follow-up is particularly noteworthy, as most existing studies on M-TEER in CRT non-responders have limited follow-up periods of one to two years [3,4].

Mitral valve transcatheter edge-to-edge repair has shown benefits in CRT non-responders, leading to improved outcomes compared to medical therapy alone [3,4]. In this specific population, right ventricular function is crucial in predicting outcomes after M-TEER. For CRT non-responders, preserved right heart function (TAPSE > 15 mm or TRPG at discharge ≤ 35 mmHg) indicates good candidates for M-TEER [11]. Our patient, a CRT non-responder with borderline right heart function (TAPSE 16.7 mm, discharge TRPG 34 mmHg), experienced long-term benefits from M-TEER. This highlights the importance of considering right ventricular function when selecting CRT non-responders for M-TEER.

The decision-making process in complex cases like ours requires a multidisciplinary approach. The heart team's thorough discussion, considering the patient's complex cardiac history, CRT non-response, and ESE findings, was crucial in recommending M-TEER. Furthermore, the procedural challenge of minimal MR under anesthesia underscores the importance of pre-procedural ESE findings in guiding clip placement. This case demonstrates that even in patients with borderline right ventricular function and complex cardiac history, M-TEER can provide significant long-term benefits when carefully selected and guided by comprehensive assessment.

## Conclusions

This case demonstrates the potential long-term benefits of M-TEER in carefully selected CRT non-responders with exercise-induced severe MR. The three-year symptomatic improvement underscores the value of comprehensive assessment, including ESE, in guiding treatment decisions for complex HFrEF patients. It highlights the importance of considering M-TEER even in cases with borderline right ventricular function when supported by a thorough evaluation. Further research is needed to optimize patient selection and evaluate the long-term outcomes of M-TEER in this challenging population.
